# Associations of Combined Exposure to Metabolic and Inflammatory Indicators with Thyroid Nodules in Adults: A Nested Case-Control Study

**DOI:** 10.1155/2024/3950894

**Published:** 2024-03-27

**Authors:** Xin-Yi Zhu, Xing-Chen Meng, Bei-Jing Cheng, Chun Wang, Jia Wang, Tian-Lin Li, Hui Li, Ke Meng, Ran Liu

**Affiliations:** ^1^The Affiliated Zhongda Hospital, Medical School of Southeast University, Nanjing, Jiangsu 210009, China; ^2^Key Laboratory of Environmental Medicine Engineering, Ministry of Education, School of Public Health, Southeast University, Nanjing, Jiangsu 210009, China

## Abstract

**Objective:**

To explore associations of combined exposure to metabolic/inflammatory indicators with thyroid nodules.

**Methods:**

We reviewed personal data for health screenings from 2020 to 2021. A propensity score matching method was used to match 931 adults recently diagnosed with thyroid nodules in a 1 : 4 ratio based on age and gender. Conditional logistic regression and Bayesian kernel machine regression (BKMR) were used to explore the associations of single metabolic/inflammatory indicators and the mixture with thyroid nodules, respectively.

**Results:**

In the adjusted models, five indicators (OR_Q4 vs. Q1_: 1.30, 95% CI: 1.07–1.58 for fasting blood glucose; OR_Q4 vs. Q1_: 1.30, 95% CI: 1.08–1.57 for systolic blood pressure; OR_Q4 vs. Q1_: 1.26, 95% CI: 1.04–1.53 for diastolic blood pressure; OR_Q4 vs. Q1_: 1.23, 95% CI: 1.02–1.48 for white blood cell; OR_Q4 vs. Q1_: 1.28, 95% CI: 1.07–1.55 for neutrophil) were positively associated with the risk of thyroid nodules, while high-density lipoproteins (OR_Q3 vs. Q1_: 0.75, 95% CI: 0.61–0.91) were negatively associated with the risk of thyroid nodules. Univariate exposure-response functions from BKMR models showed similar results. Moreover, the metabolic and inflammatory mixture exhibited a significant positive association with thyroid nodules in a dose-response pattern, with systolic blood pressure being the greatest contributor within the mixture (conditional posterior inclusion probability of 0.82). No interaction effects were found among the five indicators. These associations were more prominent in males, participants with higher age (≥40 years old), and individuals with abnormal body mass index status.

**Conclusions:**

Levels of the metabolic and inflammatory mixture have a linear dose-response relationship with the risk of developing thyroid nodules, with systolic blood pressure levels being the most important contributor.

## 1. Introduction

Thyroid nodules are overgrown masses of normal thyroid cells in the gland [1], which are classified into several types: single, multiple, solid, or cystic [2]. A previous study reported that the global prevalence of thyroid nodules has reached 4–7%, of which 8–16% turn into thyroid cancer [3]. In recent years, the prevalence of thyroid nodules in China has shown a concerning increase, with various studies reporting prevalence rates ranging from 10% to 50% [4–7]. For example, a study involving 6,985,956 participants (mean age: 42.1 ± 13.1 years) from 30 provinces and regions in China indicated an overall prevalence of thyroid nodules of 36.9% [4]. Although the rate of thyroid nodules evolving into thyroid cancer in China was similar to the findings of Burman and Wartofsky [3, 8], the high prevalence of thyroid nodules has made thyroid cancer the seventh most prevalent malignant tumor in China [9]. In addition, thyroid nodules may cause a variety of clinical sequelae such as thyroid dysfunction, dysphagia, and shortness of breath [10]. These findings suggest that thyroid nodules are a concern.

Clinical treatment of thyroid nodules is currently controversial [11]; thus, the prevention of thyroid nodules may be an effective strategy. For example, China launched a mandatory universal salt iodization program in 1996, which has been effective in controlling thyroid-related diseases [12]. However, recent studies also suggested that excessive iodine intake may increase the risk of thyroid nodules [12–14]. Thus, there is an urgent need to identify several additional modifiable risk factors, especially key factors, in adults.

Thyroid nodules are a common clinical disease caused by various factors [15]. Recent studies have shown that in addition to genetic, environmental exposure factors are more important for thyroid nodules [16]. Several environmental risk factors have been observed, including improved iodine intake [10, 17], exposure to various toxic compounds [18], and metabolic disorders and inflammatory responses [19, 20]. Metabolic disorders, including hypertension, dyslipidemia, and impaired fasting glucose, all contribute to increased thyroid nodules [21–26]. Inflammatory parameters such as white blood cell (WBC), neutrophil (N), lymphocyte (L), and monocyte (M) were significantly associated with thyroid nodules [27]. However, the exact pathophysiological pathways underlying the adverse effects of metabolic disorders and inflammatory responses on thyroid nodules are unclear. The main pathogenesis may be related to serum thyroid stimulating hormone (TSH) levels [28], which were positively associated with thyroid nodules [29]. Several studies have reported that blood pressure [30], fasting glucose [31], and inflammatory factors [8] were associated with TSH.

There were some limitations in previous studies. Firstly, most previous studies only focused on the single effects of metabolic/inflammation indicators on thyroid nodules [21, 22, 27]. To our knowledge, no study has explored the combined effects of multiple indicators. But humans often are exposed to multiple indicators simultaneously, which may have interaction effects on health [32]. Moreover, the available data are more based on cross-sectional studies and/or limited sample sizes [33]. Thus, we employed a retrospective nested case-control study utilizing a relatively large sample to explore the relationships of combined exposure to metabolic and inflammatory indicators with thyroid nodules in this study. Combining previous articles and our database, we selected eight metabolic indicators and six inflammatory indicators that may be associated with thyroid nodules [21–27]. Our study aims to (1) assess the associations of the combined exposure to eight metabolic indicators and six inflammatory indicators with the occurrence of thyroid nodules, (2) explore the indicators that may have the greatest impact on thyroid nodules in the mixture, (3) investigate interactions among mixture components, and (4) discover the susceptible subgroups.

## 2. Methods

### 2.1. Study Population

The data reported in this study came from the Health Examination Center of Zhongda Hospital affiliated with Southeast University, which was performed in Nanjing City, Jiangsu Province, China, from January 1, 2020, to December 31, 2021. Clients with severe diseases or clinical symptoms were led to the emergency or outpatient department. Thus, participants in this study were considered healthy or only have mild illnesses for physical examination. Inclusion criteria were (1) participants with health examination records in 2020 and 2021; (2) years ≥18; (3) there was no abnormal change in thyroid ultrasound in 2020; and (4) comprehensive examination of metabolic/inflammatory indicators in 2020. Exclusion criteria were (1) a history of thyroid surgery; (2) suspected Graves' disease or thyroid cancer; and (3) data missing on metabolic/inflammatory indicators in 2020. According to the inclusion and exclusion criteria, 931 adults recently diagnosed with thyroid nodules and 3724 controls (1 : 4 matched by age and gender using propensity score matching) were included in the final analysis (Figure S1). The principles of the Helsinki Declaration were followed. All data involving medical records were not publicly available, and no participants have been contacted. Ethical approval was obtained from the ethics committee of the Clinical Research Ethics Committee of Zhongda Hospital Affiliated with Southeast University (No.: 2022ZDSYLL218-P01).

### 2.2. Demographic and Anthropometric/Indicator Assessment

Demographic information was collected for the year of 2020, including gender, age, body mass index (BMI), smoking, drinking, diabetes, and hypertension. Anthropometric measurements were performed by a professionally trained nurse. Participants included in the study were required to take off their shoes, and then their height and weight were measured. In addition, participants were required to rest for at least 5–10 minutes before blood pressure measurement. Participants were asked to remain fasted from 10:00 pm the previous night and have their blood drawn by a nurse the next morning (8: 00–9: 30). A biochemical automatic analyzer (Dimension RxL Max, Siemens Corporation, German) was used to detect metabolic parameters. The whole blood count was detected by a whole blood automatic analyzer (BC-6800Plus, Mindray Medical, China).

### 2.3. Metabolic and Inflammatory Indicators

Combining previous articles and our database, we selected eight metabolic indicators and six inflammatory indicators of 2020. Metabolic indicators included total cholesterol (TC), low-density lipoprotein (LDL), triglyceride (TG), high-density lipoprotein (HDL), fasting blood glucose (FBG), uric acid (UA), systolic blood pressure (SBP), and diastolic blood pressure (DBP). Inflammatory parameters included WBC, M, basophils (B), eosinophils (E), L, and N.

### 2.4. Definition of Thyroid Nodules

Thyroid ultrasonography is performed by experienced sonographers using a high-frequency probe. Thyroid nodules have been defined as any solid (including solid with cystic component) and nodular lesion, which are different from the adjacent parenchyma in the thyroid gland by ultrasonography [15]. Laboratory technicians were trained by technical support staff to use the machines for analysis and to calibrate the analyzers according to standard quality assurance protocols.

### 2.5. Potential Covariates

Confounding factors were considered as gender (male and female), age (<30, 30–39, 40–49, and ≥50 years old), diabetes (no and yes), hypertension (no and yes), smoking (no and yes), drinking (no and yes), and BMI (BMI<18.5, 18.5–23.9, 24–27.9, and >28 kg/m^2^). Diabetes was identified by a FBG level of ≥7.0 mmol/L (126 mg/dL). Hypertension was identified by a SBP of ≥140 mmHg or a DBP of ≥90 mmHg.

### 2.6. Statistical Analysis

The characteristics and metabolic/inflammatory indicators across two groups (thyroid nodules and nonthyroid nodules) were compared using the chi-square test for categorical variables and *t*-tests for continuous variables. Spearman's correlation coefficient was used to assess the correlations between the indicators measured at the baseline. In addition, intraclass correlation coefficients (ICCs) were used to explore the correlations between indicators measured at 2020 and 2021. In the following regression models, we used a zero-mean normalization approach to standardize metabolic and inflammatory indicators. The histograms showed that most of the indicators after zero-mean normalization were normally or approximately normally distributed (Figure S2).

Multivariate conditional logistic regression models were employed to evaluate associations between multiple indicators and thyroid nodules. Participants were categorized into quartiles based on the level of each indicator, and the lowest quartile was used as a reference. Adjusted odds ratio (OR) and 95% confidence interval (CI) were calculated for the occurrence of thyroid nodules. The covariates included age, gender, diabetes, and hypertension. The model was adjusted for matching variables to account for residual confounding. False discovery rate (FDR) corrections were used to adjust *p* values. Since there were many indicators in this study, we screened five indicators (HDL, FBG, SBP, WBC, and N) based on the above regression results and correlation coefficients (Figure S3) and then included them in the subsequent BKMR models. Given the nonlinear and interactive effects, BKMR analysis was performed to assess the combined effects of multiple metabolic and inflammatory indicators [32]. The models were executed up to 10,000 iterations using a Markov chain Monte Carlo algorithm [34]. Five indicators were classified into two groups based on metabolic and inflammatory indicators. We selected the key indicators for thyroid nodules by calculating the group posterior inclusion probability (groupPIP) and conditional posterior inclusion probability (condPIP), where the threshold value of PIP was 0.5 [35]. The results of BKMR analysis were as follows: (1) nonlinear and/or nonadditive associations of individual indicators with the risk of thyroid nodules, (2) joint effects of the indicator mixture on the risk of thyroid nodules, (3) the relative importance of individual indicators within the mixture, and (4) interactive effects among mixture components.

Stratified analyses according to gender (male, female), age (<40, ≥40 years), and BMI (18.5–23.9, <18.5/>23.9 kg/m^2^) were conducted. Furthermore, we conducted three sensitivity analyses to evaluate the stability of results. First, given the possible confounding and mediating effects of BMI in these associations, BMI was not adjusted in the formal analysis. In the sensitivity analysis, we examined the potential confounding effect of BMI by adding the BMI variable to the BKMR model. Although smoking and drinking are risk factors for thyroid nodules, there is a large amount of missing data for these two factors in this study. We grouped the missing values of smoking or drinking into a category. In the second sensitivity analysis, we examined the potential confounding effect of two factors (dummy variables) by adding them to the BKMR model. Finally, all metabolic and inflammatory indicators were included in the BKMR model, and the covariates were controlled for age, gender, diabetes, and hypertension. SPSS (version 20; IBM SPSS Statistics) and R (version 4.0.2; R Foundation for Statistical Computing) were used to conduct statistical analysis. Two-sided *P* values below 0.05 were considered statistically significant.

## 3. Results

### 3.1. Characteristics of the Study Population

The characteristics of the participants are presented in Table 1. Of all participants, 61.2% were female and 57.3% were aged 40 years and above. A total of 931 individuals were newly diagnosed with thyroid nodules during the study period. The median maximum diameter of thyroid nodules was 0.30 (interquartile range: 0.24–0.40) mm (Figure S4). Comparisons of the risk of thyroid nodules across groups are also shown in Table 1. Participants with thyroid nodules were more likely to have higher BMI, diabetes, and hypertension than those with nonthyroid nodules. In addition, participants with thyroid nodules had higher levels of LDL, FBG, SBP, DBP, WBC, and N, whereas no significant differences were found for other indicators (Table 1).

Spearman's correlation coefficients between metabolic/inflammatory indicators are displayed in Table S1. Spearman's correlation coefficients ranged from −0.010 to 0.890, with the highest correlation coefficient between TC and LDL (*r* = 0.888), and the remaining ones in descending order were SBP and DBP (*r* = 0.787) and WBC and M (*r* = 0.663). In addition, Table S2 showed that the correlations between all indicators for 2020 and 2021 were moderate to strong (ICCs: 0.520–0.839).

### 3.2. Single Indicator Exposure and Thyroid Nodules

The associations of metabolic and inflammatory indicators with thyroid nodules are shown in Table S3 and Table 2. After adjusting for age, gender, diabetes, and hypertension, the models showed that five indicators (FBG, SBP, DBP, WBC, and N) had significant positive associations with thyroid nodules (*P* value <0.05), but HDL had a significant negative association (*P* value <0.05). For example, compared with participants in the lowest quartile of FBG, SBP, DBP, WBC, and N, participants in the highest quartile showed 30% (95% CI: 1.07, 1.58), 30% (95% CI: 1.08, 1.57), 26% (95% CI: 1.04, 1.53), 23% (95% CI: 1.02, 1.48), and 28% (95% CI: 1.07, 1.55) increased the risk of thyroid nodules, respectively. Moreover, compared with participants in the lowest quartile of HDL, participants in the 3^rd^ quartile showed a 25% (95% CI: 0.61, 0.91) decreased the risk of thyroid nodules. After FDR adjustments were made, similar results of statistically significant were found. Since there were many indicators in this study, we screened five indicators (HDL, FBG, SBP, WBC, and N) based on the above regression results and correlation coefficients (Figure S3) and then included them in the subsequent BKMR models.

### 3.3. BKMR Analyses

**Figure 1:**
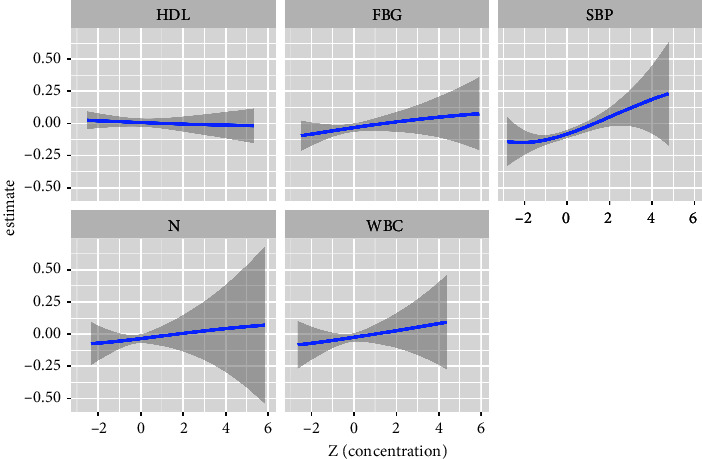
Univariate exposure-response functions and 95% confidence intervals for associations between single metabolic/inflammatory indicators and the risk of thyroid nodules when other indicators were fixed at the median. Adjusted variables included age, gender, diabetes, and hypertension.

**Figure 2:**
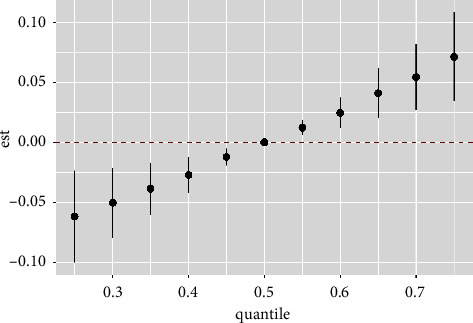
The overall effect of the mixture (95% confidence intervals) when all of the indicators were fixed at a specific quantile (ranging from the 25th percentile to 75th percentile), as compared to when all indicators were fixed at their median values (the 50th percentile). Adjusted variables included age, gender, diabetes, and hypertension.

**Figure 3:**
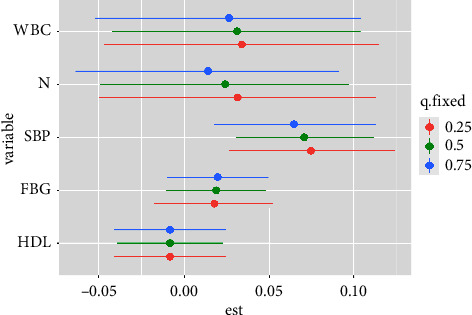
Single-exposure effects (95% confidence intervals), defined as the changes in the risk of thyroid nodules associated with a change in a particular indicator from its 25th to its 75th percentile, where all of the remaining indicators were fixed at a specific quantile (the 25th, 50th, or 75th percentile). Adjusted variables included age, gender, diabetes, and hypertension.

**Figure 4:**
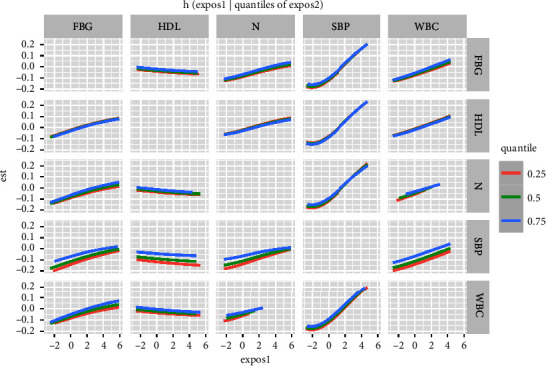
Bivariate exposure response functions. Each cell represented the exposure-response curve for the column indicator when the row indicator was fixed at 10th, 50th, and 90th percentiles and the remaining indicators were fixed at their medians.

Figure 1 also showed linear relationships between exposure to single indicators and thyroid nodules when other indicators' exposure was fixed at the median.  Figure 2 showed that a significant joint effect of the five indicators was found when all indicators were at or above their 55th percentile compared to the median. In addition, a slightly decreased and positive association between SBP and thyroid nodules was found when the other four indicators were fixed at different percentiles (25th, 50th, or 75th) (Figure 3). SBP exhibited strong linear associations, which was supported by PIPs in Table 3. One groupPIPs were higher than 0.5, and the condPIP of SBP (0.82) was the highest in the group. Finally, we estimated bivariate exposure-response functions for the five indicators (Figure 4). We did not find a significant interaction effect among the five indicators.

### 3.4. Subgroup and Sensitivity Analysis

The stratified analyses by gender, age, and BMI showed that these joint associations were more obvious in males, participants with higher age (≥40 years old), and individuals with abnormal BMI (Figures S5–S10). Besides, after controlling for BMI (continuous), smoking and drinking, or all 14 indicators, three sensitivity analyses did not materially change our findings (Figures S11–S13).

## 4. Discussion

### 4.1. Key Findings

In the adjusted model, FBG, SBP, DBP, WBC, and N were significantly positively correlated with thyroid nodules compared to their lowest concentration groups, while HDL was significantly negatively correlated. Univariate exposure-response functions from BKMR models showed similar results. Moreover, our study found a linear dose-response relationship between the mixture of metabolic and inflammatory indicators and thyroid nodules, and SBP was the most important contributor within the mixture. Nevertheless, no interaction effects were found among the five indicators. These associations were more prominent in males, participants with higher age (≥40 years old), and individuals with abnormal BMI. To our knowledge, this is the first study to examine associations of combined exposure to metabolic and inflammatory indicators with thyroid nodules.

### 4.2. Metabolic and Inflammatory Indicators

Consistent with the results of most previous studies [4, 33], our study also showed that blood pressure was positively associated with thyroid nodules. A recent meta-analysis [33] showed that abnormal blood pressure was associated with thyroid nodules (OR = 1.68, 95% CI: 1.62–1.75). Several large-scale studies, such as [36, 37], have concluded that abnormal blood pressure is a risk factor for thyroid nodules. Other studies [26, 38] with small samples have yielded inconsistent results. The exact mechanism of the risk of thyroid nodules due to hypertension is not known. Several studies have shown a positive correlation between TSH and SBP/DBP [39, 40], and high TSH levels in hypertensive patients may contribute to the formation of thyroid nodules. In addition, we cannot ignore the possibility of the potential confounding effect of TSH on the association between blood pressure and thyroid nodules. Unfortunately, only 30% of the individuals in this study underwent TSH measurement. This severe selection bias hindered the possibility of meaningful mediation analyses, and thus, further studies are warranted to clarify the underlying biological mechanisms.

Consistent with most previous studies [33, 41–44], we found a significant positive association between blood glucose and thyroid nodules. Recently, a meta-analysis [33] also showed that hyperglycemia was associated with thyroid nodules (OR = 1.59, 95% CI: 1.46–1.74). One possible explanation is the confounding effects of insulin resistance. On the one hand, some studies have shown that insulin resistance can promote the formation and growth of thyroid nodules [33, 45]; on the other hand, insulin resistance is a key factor in the pathogenesis of impaired glucose metabolism [46]. Regrettably, no data on insulin resistance were collected in this study, so the confounding effects of insulin resistance could not be ruled out. As we know, high levels of TSH can lead to the development of thyroid nodules [47, 48]. A study [41] has shown higher serum TSH levels in serum type 2 diabetic patients than in control prediabetic and control patients, providing another possible explanation.

In this study, higher levels of HDL were significantly negatively associated with thyroid nodules, which was consistent with the limited studies [4, 26]. A cross-sectional study showed that elevated HDL levels were negatively correlated with thyroid nodules, while TG and LDL were positively correlated [4]. Another case-control study also showed a significant association between low HDL (OR = 2.77, 95% CI: 1.44–5.30) and thyroid nodules. Compared to the previous two studies [4, 26], we used a retrospective nested case-control to provide relatively reliable evidence. However, the underlying mechanism by which high levels of serum HDL reduce the development of thyroid nodules remains unclear. Further studies are needed to examine the prospective association of HDL with thyroid nodules and to better understand the mechanisms.

Our retrospective nested case-control study showed that WBC and N increased the risk of thyroid nodules. Li et al. discovered a higher prevalence of thyroid nodules in participants with high levels of inflammation (WBC, N, L, and M) by using propensity score matching for metabolic parameters and other confounding factors [8]. Moreover, a retrospective cohort study (included 6587 participants) showed that M was a risk factor for thyroid nodules [48]. Haider et al. also reported that the monocyte-to-high-density lipoprotein cholesterol ratio (MHR) and neutrophil-to-lymphocyte ratio (NLR) were significantly associated with the presence of thyroid nodules [49]. These findings are consistent with our expectations. As we know, chronic inflammation plays a role in the development of thyroid nodules [50].

### 4.3. Combined Exposure

Considering the high correlation and complexity between indicators, traditional methods may not provide a true view of the relationship between mixed exposures to multiple indicators and thyroid nodules. However, to our knowledge, no epidemiological studies are addressing this issue. In this study, we used the BKMR model to assess associations of combined exposure to multiple indicators with thyroid nodules. First, consistent with our expectations, we found a linear dose-response relationship between the combined five metabolic/inflammatory indicators (HDL, FBG, SBP, WBC, and N) and the risk of thyroid nodules. Although both metabolic and inflammatory indicators are thought to have an impact on thyroid nodules, their relative importance remains unclear. In this study, we found that metabolic indicators were more important than inflammatory indicators. Within this mixture, blood pressure is the most important component. Our findings suggest that controlling metabolic indicators, especially blood pressure, may be important in reducing the risk of thyroid nodules in adults.

### 4.4. Subgroup Analysis

Identifying susceptible populations is important for both public health and clinical practice; however, knowledge in this area remains unclear. Most studies have shown a higher prevalence of thyroid nodules in females than in males [4, 22, 51], but few studies have explored the gender-specific associations between metabolic/inflammatory indicators and thyroid nodules [22]. We found that the association of joint exposure was more prominent in males. In addition, we also found that blood pressure was the main determinant in males and blood glucose in females. Ding et al. found that diabetes (OR = 1.47, 95% CI: 1.17–1.84) remained strongly and independently associated with a higher risk of thyroid nodules in females but not in males. In a retrospective cohort study, Huang et al. found that the association between the metabolic indicator (uric acid) and thyroid nodules was more pronounced in females [48]. Although limited results of the gender-specific association between metabolic indicators and thyroid nodules are controversial, all these results supported the fact that gender may play a moderating role in this association. It is still too early to draw any conclusions on the gender difference in the relationship between metabolic indicators and thyroid nodules, and further studies are warranted. Interestingly, we also found that associations of joint exposure were more prominent in participants with higher age (≥40 years old) and individuals with abnormal body mass index status, which suggested potential harmful effects of high age, under- or overweight status.

### 4.5. Implications for Public Health

Our findings may have implications for public health. The linear dose-response relationship of the mixture of metabolic and inflammatory indicators with thyroid nodules provides valuable insights. Notably, our findings highlight the dominant role of SBP in the mixture. This provides key clues for customizing prevention strategies, suggesting that focusing on managing SBP and considering a combination of approaches (e.g., appropriate medication use and exercise interventions) to intervene with metabolic and inflammatory factors. Importantly, our study reveals population-specific patterns in the observed associations. Males, individuals aged 40 years and older, and those with abnormal levels of BMI showed stronger associations, providing targeted information to optimize prevention strategies. This detailed understanding allows for the development of more targeted interventions for different populations. For example, for males and individuals with higher age, we can emphasize the critical role of SBP and encourage regular blood pressure monitoring and active blood pressure management. Meanwhile, for individuals with abnormal BMI, we recommend weight management and nutritional education programs to help keep their metabolism and inflammation in balance. These specific measures are expected to increase public awareness of health and motivate more people to adopt active lifestyles, thereby reducing the risk of thyroid nodules.

### 4.6. Limitations and Strengths

There are three strengths of this study. First, to our knowledge, this is the first study to examine the associations of combined exposure to metabolic/inflammatory indicators with thyroid nodules. Second, utilizing nested case-control studies helps mitigate selection bias, recall bias, and confounding bias, thereby enhancing the internal validity of associations. Third, we performed a series of subgroup and sensitivity analyses to show that the results were considerably robust.

However, this study also has several limitations. First, the study population was from one health examination center, which led to possible limitations in the generalization of our findings to other regions or the general population. Future studies could include data from multiple medical centers or different population characteristics to further validate our results. Second, it is important to note that because of the observational research design, we can only infer correlation, not causation. Third, one-time sample measures may bias internal exposure estimates. We explored the association between baseline and follow-up indicators and found moderate to strong reproducibility for these indicators (ICCs ranged from 0.520 to 0.839). Thus, we believe that one-time sample measures may reflect the long-term exposure levels to a certain extent. Fourth, we followed up for only one year, which was unlikely to affect our overall conclusions but limited our ability to assess different thyroid grades. For example, the median maximum diameter of thyroid nodules in this study was only 0.30 (interquartile range: 0.24–0.40) mm. Fifth, general several inflammatory indicators (e.g., C-reactive protein and interleukin-6) were not measured. Therefore, these clinical indicators could not be considered in this analysis, which may underestimate the association of combined exposure to inflammatory indicators with thyroid nodules. Finally, since our data came from the health examination center, some confounding factors were not collected well. For example, there was a large amount of missing smoking and drinking, and we could only adjust for these factors in the sensitivity analysis. In addition, residual confounding of unmeasured variables (e.g., physical activities, dietary structure, and iodine content) cannot be excluded.

## 5. Conclusions

Our study found a linear dose-response relationship between the mixture of metabolic/inflammatory indicators and thyroid nodules, and SBP was the most important contributor within the mixture. These associations were more prominent in males, participants with higher age (≥40 years old), and individuals with abnormal BMI. Our findings suggest that reduced metabolic and inflammatory levels, especially reduced blood pressure levels, may be important in preventing thyroid nodules. Further studies are needed to explore the prospective association between metabolic/inflammatory indicators and thyroid nodules and to elucidate the complex mechanisms between these indicators and thyroid nodules.

## Figures and Tables

**Table 1:** Characteristics and metabolic/inflammatory indicators of the study population.

Variables	Total	Thyroid nodules	Nonthyroid nodules	*χ* ^2^ */t*	*P* value
Total (%)	4655	931 (20.00)	3724 (80.00)		
Gender (%)				0.08	0.775
Male	1806 (38.80)	362 (20.20)	1441 (79.80)		
Female	2849 (61.20)	566 (19.90)	2283 (80.10)		
Age (years, %)				2.02	0.569
<30	722 (15.50)	146 (20.20)	576 (79.80)		
30–39	1265 (27.20)	238 (18.80)	1027 (81.20)		
40–49	1234 (26.50)	246 (19.90)	988 (80.10)		
≥50	1434 (30.80)	301 (21.00)	1133 (79.00)		
BMI (kg/m^2^, %)				11.78	**0.008**
<18.5	227 (4.90)	33 (14.50)	194 (85.50)		
18.5–23.9	2525 (54.20)	477 (18.90)	2048 (81.10)		
24.0–27.9	1503 (32.30)	336 (22.40)	1167 (77.60)		
≥28.0	400 (8.60)	85 (21.30)	315 (78.80)		
Smoking (%)^ab^					
No	1292 (27.80)	245 (19.00)	1047 (81.00)	2.10	0.350
Yes	372 (8.00)	69 (18.50)	303 (81.50)		
Missing	2991 (64.30)	617 (20.60)	2374 (79.40)		
Drinking (%)^ab^					
No	1133 (24.30)	219 (19.30)	914 (80.70)	7.68	0.422
Yes	528 (11.30)	95 (18.00)	433 (82.00)		
Missing	2994 (63.20)	617 (20.60)	2377 (79.40)		
Diabetes (%)^c^				4.99	**0.025**
No	4504 (96.80)	890 (19.80)	3614 (80.20)		
Yes	151 (3.20)	41 (27.20)	110 (72.80)		
Hypertension (%)^d^				7.41	**0.006**
No	3591 (77.10)	687 (19.10)	2904 (80.90)		
Yes	1064 (22.90)	244 (22.90)	820 (77.10)		
Metabolic indicators					
TC (mmol/L)	4.82 ± 0.89	4.87 ± 0.94	4.81 ± 0.88	−1.81	0.070
LDL (mmol/L)	2.73 ± 0.67	2.77 ± 0.69	2.71 ± 0.66	−2.22	**0.027**
TG (mmol/L)	1.39 ± 0.94	1.44 ± 0.96	1.38 ± 0.96	−1.80	0.072
HDL (mmol/L)	1.44 ± 0.29	1.43 ± 0.29	1.45 ± 0.29	1.46	0.144
FBG (mmol/L)	5.26 ± 0.76	5.31 ± 0.77	5.24 ± 0.76	−2.50	**0.013**
UA (mmol/L)	332.80 ± 87.37	335.89 ± 87.73	332.03 ± 87.27	−1.20	0.228
SBP (mmHg)	125.06 ± 18.35	126.84 ± 18.84	124.61 ± 18.20	−3.31	**0.001**
DBP (mmHg)	75.67 ± 11.80	76.75 ± 11.90	75.40 ± 11.76	−3.13	**0.002**
Inflammatory indicators					
WBC (10^9^ cells/L)	6.08 ± 1.47	6.20 ± 1.46	6.05 ± 1.47	−2.65	**0.008**
M (10^9^ cells/L)	0.35 ± 0.11	0.35 ± 0.11	0.34 ± 0.11	−1.88	0.060
B (10^9^ cells/L)	0.03 ± 0.02	0.03 ± 0.02	0.03 ± 0.02	−1.26	0.270
E (10^9^ cells/L)	0.14 ± 0.11	0.14 ± 0.11	0.14 ± 0.11	0.51	0.613
L (10^9^ cells/L)	1.99 ± 0.57	2.02 ± 0.55	2.00 ± 0.57	−1.68	0.092
N (10^9^ cells/L)	3.58 ± 1.12	3.66 ± 1.12	3.56 ± 1.12	−2.47	**0.013**

BMI: body mass index; TC: total cholesterol; LDL: low-density lipoprotein; TG: triglycerides; HDL: high-density lipoprotein; FBG: fasting blood glucose; UA: uric acid; SBP: systolic blood pressure; DBP: diastolic blood pressure; WBC: white blood cell; M: monocyte; B: basophil; E: eosinophil; L: lymphocyte; N: neutrophil. ^a^Due to the missing covariate data, subgroup totals may not sum to the total sample population. ^b^The chi-square values were calculated without including missing values. ^c^Diabetes was identified by a fasting blood glucose level of ≥7.0 mmol/L (126 mg/dL). ^d^Hypertension was identified by a SBP of ≥140 mmHg or a DBP of ≥90 mmHg. Bold values indicate statistical significance, *P* < 0.05.

**Table 2:** Associations between metabolic/inflammatory indicators and thyroid nodules using conditional logistic regression.

Metabolic indicators	Adjusted models^a^		
	OR (95% CI)	*P* value	*P* value^b^

*TC*			
Q1	1	—	—
Q2	1.08 (0.90, 1.30)	0.413	0.707
Q3	1.07 (0.89, 1.29)	0.471	0.707
Q4	1.01 (0.84, 1.22)	0.883	0.883
*P*-trend	0.956		

*LDL*			
Q1	1	—	—
Q2	1.12 (0.92, 1.36)	0.244	0.453
Q3	1.11 (0.91, 1.34)	0.302	0.453
Q4	1.06 (0.87, 1.28)	0.583	0.583
*P*-trend	0.674		

*TG*			
Q1	1	—	—
Q2	0.99 (0.82, 1.20)	0.933	0.933
Q3	1.10 (0.91, 1.34)	0.307	0.461
Q4	1.15 (0.94, 1.40)	0.164	0.461
*P*-trend	0.102		

*HDL*			
Q1	1	—	—
Q2	0.83 (0.68, 1.00)	0.056	0.084
Q3	0.75 (0.61, 0.91)	**0.005**	**0.015**
Q4	0.87 (0.71, 1.06)	0.162	0.162
*P*-trend	0.188		

*FBG*			
Q1	1	—	—
Q2	1.10 (0.90, 1.33)	0.348	0.348
Q3	1.20 (1.00, 1.45)	0.056	0.084
Q4	1.30 (1.07, 1.58)	**0.008**	**0.024**
*P*-trend	**0.005**		

*UA*			
Q1	1	—	—
Q2	1.03 (0.85, 1.24)	0.795	0.795
Q3	1.10 (0.89, 1.36)	0.363	0.626
Q4	1.10 (0.88, 1.37)	0.417	0.626
*P*-trend	0.359		

*SBP*			
Q1	1	—	—
Q2	0.96 (0.79, 1.17)	0.692	0.865
Q3	0.98 (0.81, 1.19)	0.865	0.865
Q4	1.30 (1.08, 1.57)	**0.006**	**0.018**
*P*-trend	**0.004**		

*DBP*			
Q1	1	—	—
Q2	0.99 (0.81, 1.20)	0.916	0.916
Q3	1.09 (0.90, 1.31)	0.369	0.554
Q4	1.26 (1.04, 1.53)	**0.016**	**0.048**
*P*-trend	**0.009**		

Inflammatory indicators	Adjusted models^a^		
	OR (95% CI)	*P* value	*P* value^b^

*WBC*			
Q1	1	—	—
Q2	1.03 (0.86, 1.25)	0.724	0.724
Q3	1.23 (1.03, 1.48)	**0.026**	**0.044**
Q4	1.23 (1.02, 1.48)	**0.029**	**0.044**
*P*-trend	0.095		

*M*			
Q1	1	—	—
Q2	1.01 (0.84, 1.20)	0.955	0.955
Q3	1.09 (0.89, 1.32)	0.406	0.609
Q4	1.20 (0.99, 1.44)	0.062	0.186
*P*-trend	0.057		

*L*			
Q1	1	—	—
Q2	1.16 (0.97, 1.39)	0.111	0.167
Q3	1.01 (0.83, 1.21)	0.957	0.957
Q4	1.19 (0.99, 1.43)	0.061	0.167
*P*-trend	0.625		

*B*			
Q1	1	—	—
Q2	0.95 (0.79, 1.14)	0.564	0.564
Q3	1.17 (0.97, 1.40)	0.104	0.312
Q4	1.08 (0.89, 1.30)	0.438	0.564
*P*-trend	0.256		

*E*			
Q1	1	—	—
Q2	1.07 (0.89, 1.30)	0.463	0.753
Q3	1.06 (0.87, 1.29)	0.547	0.753
Q4	0.97 (0.79, 1.18)	0.753	0.753
*P*-trend	0.311		

*N*			
Q1	1	—	—
Q2	1.04 (0.86, 1.26)	0.683	0.683
Q3	1.25 (1.04, 1.50)	**0.020**	**0.030**
Q4	1.28 (1.07, 1.55)	**0.008**	**0.024**
*P*-trend	**0.040**		

TC: total cholesterol; LDL: low-density lipoprotein; TG: triglycerides; HDL: high-density lipoprotein; FBG: fasting blood glucose; UA: uric acid; SBP: systolic blood pressure; DBP: diastolic blood pressure; WBC: white blood cell; M: monocyte; B: basophil; E: eosinophil; L: lymphocyte; N: neutrophil. ^a^Adjusting age, gender, diabetes, and hypertension. ^b^False discovery rate (Benjamini and Hochberg). Bold values indicate statistical significance, *P* < 0.05.

**Table 3:** PIPs for group inclusion and conditional inclusion into thyroid nodules using Bayesian kernel machine regression (BKMR) models.

Variables	Group	groupPIP	condPIP
HDL	1	0.84	0.02
FBG	1	0.84	0.16
SBP	1	0.84	0.82
N	2	0.48	0.54
WBC	2	0.48	0.46

PIP: Posterior inclusion probabilities; HDL: high-density lipoprotein; FBG: fasting blood glucose; SBP: systolic blood pressure; WBC: white blood cell; N: neutrophil.

## Data Availability

The datasets used and/or analyzed during the current study are available from the corresponding author on reasonable request. All data involving medical records were not publicly available, and no participants have been contacted.

## Supplementary Materials

Supplementary Materials Supplementary 1. Table S1: correlations between metabolic/inflammatory indicators using Spearman's correlation analysis in 2020. Supplementary 2. Table S2: intraclass correlation coefficients of metabolic/inflammatory indicators in 2020 and 2021. Supplementary 3. Table S3: associations between metabolic/inflammatory indicators and thyroid nodules using conditional logistic regression. Supplementary 4. Figure S1: flow chart. Supplementary 5. Figure S2: the histograms of metabolic/inflammatory indicators. Supplementary 6. Figure S3: correlations between metabolic/inflammatory indicators using Spearman's correlation analysis. Supplementary 7. Figure S4: maximum diameter of thyroid nodules (mm) in 931 participants. Supplementary 8. Figure S5: associations between five metabolic/inflammatory indicators and the risk of thyroid nodules in male, estimated using Bayesian kernel machine regression (BKMR). Adjusted variables included age, diabetes, and hypertension. Supplementary 9. Figure S6: associations between five metabolic/inflammatory indicators and the risk of thyroid nodules in female, estimated using Bayesian kernel machine regression (BKMR). Adjusted variables included age, diabetes, and hypertension. Supplementary 10. Figure S7: associations between five metabolic/inflammatory indicators and the risk of thyroid nodules in participants with lower age (<40 years old), estimated using Bayesian kernel machine regression (BKMR). Adjusted variables included gender, diabetes, and hypertension. Supplementary 11. Figure S8: associations between five metabolic/inflammatory indicators and the risk of thyroid nodules in participants with lower age (≥40 years old), estimated using Bayesian kernel machine regression (BKMR). Adjusted variables included gender, diabetes, and hypertension. Supplementary 12. Figure S9: associations between five metabolic/inflammatory indicators and the risk of thyroid nodules in participants with normal body mass index status (18.5–23.9 kg/m^2^), estimated using Bayesian kernel machine regression (BKMR). Adjusted variables included gender, age, diabetes, and hypertension. Supplementary 13. Figure S10: associations between five metabolic/inflammatory indicators and the risk of thyroid nodules in participants with abnormal body mass index status (<18.5 or >23.9 kg/m^2^), estimated using Bayesian kernel machine regression (BKMR). Adjusted variables included gender, age, diabetes, and hypertension. Supplementary 14. Figure S11: associations between five metabolic/inflammatory indicators and the risk of thyroid nodules in adults, estimated using Bayesian kernel machine regression (BKMR). Adjusted variables included gender, age, diabetes, hypertension, and BMI (continuous). Supplementary 15. Figure S12: associations between five metabolic/inflammatory indicators and the risk of thyroid nodules in adults, estimated using Bayesian kernel machine regression (BKMR). Adjusted variables included gender, age, diabetes, hypertension, smoking, and drinking. Supplementary 16. Figure S13: associations between 14 metabolic/inflammatory indicators and the risk of thyroid nodules in adults, estimated using Bayesian kernel machine regression (BKMR). Adjusted variables included gender, age, diabetes, and hypertension.
