# Supplementary material for: Associations of Combined Exposure to Metabolic and Inflammatory Indicators with Thyroid Nodules in Adults: A Nested Case-Control Study
Source: Int J Endocrinol. 2024 Mar 27;2024:3950894. doi: 10.1155/2024/3950894 (PMC10990645; doi:10.1155/2024/3950894)
Supplement: Supplementary Materials — Supplementary 1. Table S1: correlations between metabolic/inflammatory indicators using Spearman's correlation analysis in 2020. Supplementary 2. Table S2: intraclass correlation coefficients of metabolic/inflammatory indicators in 2020 and 2021. Supplementary 3. Table S3: associations between metabolic/inflammatory indicators and thyroid nodules using conditional logistic regression. Supplementary 4. Figure S1: flow chart. Supplementary 5. Figure S2: the histograms of metabolic/inflammatory indicators. Supplementary 6. Figure S3: correlations between metabolic/inflammatory indicators using Spearman's correlation analysis. Supplementary 7. Figure S4: maximum diameter of thyroid nodules (mm) in 931 participants. Supplementary 8. Figure S5: associations between five metabolic/inflammatory indicators and the risk of thyroid nodules in male, estimated using Bayesian kernel machine regression (BKMR). Adjusted variables included age, diabetes, and hypertension. Supplementary 9. Figure S6: associations between five metabolic/inflammatory indicators and the risk of thyroid nodules in female, estimated using Bayesian kernel machine regression (BKMR). Adjusted variables included age, diabetes, and hypertension. Supplementary 10. Figure S7: associations between five metabolic/inflammatory indicators and the risk of thyroid nodules in participants with lower age (<40 years old), estimated using Bayesian kernel machine regression (BKMR). Adjusted variables included gender, diabetes, and hypertension. Supplementary 11. Figure S8: associations between five metabolic/inflammatory indicators and the risk of thyroid nodules in participants with lower age (≥40 years old), estimated using Bayesian kernel machine regression (BKMR). Adjusted variables included gender, diabetes, and hypertension. Supplementary 12. Figure S9: associations between five metabolic/inflammatory indicators and the risk of thyroid nodules in participants with normal body mass index status (18.5–23.9 kg/m2), esti [file 3950894.f1.docx]

**Supplementary materials**

**Associations of combined exposure to metabolic and inflammatory indicators with thyroid nodules in adults: a nested case–control study**

**Authors:** Xin-Yi Zhu^ab^, Xing-Chen Meng^b^, Bei-Jing Cheng^b^, Chun Wang^b^, Jia Wang^b^, Tian-Lin Li^b^, Hui Li^b^, Ke Meng^b^, Ran Liu^b^*

^a^ The Affiliated Zhongda Hospital, Medical School of Southeast University, Nanjing, Jiangsu 210009, China.

^b^ Key Laboratory of Environmental Medicine Engineering, Ministry of Education, School of Public Health, Southeast University, Nanjing, Jiangsu 210009, China.

**Corresponding author:**

Ran Liu, Key Laboratory of Environmental Medicine Engineering, Ministry of Education, School of Public Health, Southeast University, 87 Dingjiaqiao Street, Nanjing, Jiangsu.

Tel: +86 13813373523; Fax: none; E-mail: [ranliu@seu.edu.cn](mailto:ranliu@seu.edu.cn)

**Table S1** Correlations between metabolic/inflammatory indicators using spearman’s correlation analysis in 2020

**Table S2** Intraclass correlation coefficients of metabolic/ inflammatory indicators in 2020 and 2021

**Table S3** Associations between metabolic/inflammatory indicators and thyroid nodules using conditional logistic regression

**Figure S1.** Flow chart

**Figure S2.** The histograms of metabolic/inflammatory indicators

**Figure S3.** Correlations between metabolic/inflammatory indicators using spearman’s correlation analysis

**Figure S4.** Maximum diameter of thyroid nodules (mm) in 931 participants

**Figure S5.** Associations between five metabolic/inflammatory indicators and the risk of thyroid nodules in male, estimated using Bayesian kernel machine regression (BKMR). Adjusted variables included age, diabetes, and hypertension.

**Figure S6.** Associations between five metabolic/inflammatory indicators and the risk of thyroid nodules in female, estimated using Bayesian kernel machine regression (BKMR). Adjusted variables included age, diabetes, and hypertension.

**Figure S7.** Associations between five metabolic/inflammatory indicators and the risk of thyroid nodules in participants with lower age (<40 years old), estimated using Bayesian kernel machine regression (BKMR). Adjusted variables included gender, diabetes, and hypertension.

**Figure S8.** Associations between five metabolic/inflammatory indicators and the risk of thyroid nodules in participants with lower age (≥ 40 years old), estimated using Bayesian kernel machine regression (BKMR). Adjusted variables included gender, diabetes, and hypertension.

**Figure S9.** Associations between five metabolic/inflammatory indicators and the risk of thyroid nodules in participants with normal body mass index status (18.5–23.9 kg/m2), estimated using Bayesian kernel machine regression (BKMR). Adjusted variables included gender, age, diabetes, and hypertension.

**Figure S10.** Associations between five metabolic/inflammatory indicators and the risk of thyroid nodules in participants with abnormal body mass index status (<18.5 or >23.9 kg/m2), estimated using Bayesian kernel machine regression (BKMR). Adjusted variables included gender, age, diabetes, and hypertension.

**Figure S11.** Associations between five metabolic/inflammatory indicators and the risk of thyroid nodules in adults, estimated using Bayesian kernel machine regression (BKMR). Adjusted variables included gender, age, diabetes, hypertension, and BMI (continuous).

**Figure S12.** Associations between five metabolic/inflammatory indicators and the risk of thyroid nodules in adults, estimated using Bayesian kernel machine regression (BKMR). Adjusted variables included gender, age, diabetes, hypertension, smoking, and drinking.

**Figure S13.** Associations between 14 metabolic/inflammatory indicators and the risk of thyroid nodules in adults, estimated using Bayesian kernel machine regression (BKMR). Adjusted variables included gender, age, diabetes, and hypertension.

**Table S1** Correlations between metabolic/inflammatory indicators using spearman’s correlation analysis in 2020

|  | TC | LDL | TG | HDL | FBG | UA | SBP | DBP | WBC | M | B | C | L | N |
| --- | --- | --- | --- | --- | --- | --- | --- | --- | --- | --- | --- | --- | --- | --- |
| TC | 1.000 |  |  |  |  |  |  |  |  |  |  |  |  |  |
| LDL | 0.888** | 1.000 |  |  |  |  |  |  |  |  |  |  |  |  |
| TG | 0.310** | 0.337** | 1.000 |  |  |  |  |  |  |  |  |  |  |  |
| HDL | 0.384** | 0.156** | -0.425** | 1.000 |  |  |  |  |  |  |  |  |  |  |
| FBG | 0.131** | 0.124** | 0.230** | -0.149** | 1.000 |  |  |  |  |  |  |  |  |  |
| UA | 0.110** | 0.240** | 0.425** | -0.344** | 0.140** | 1.000 |  |  |  |  |  |  |  |  |
| SBP | 0.134** | 0.134** | 0.267** | -0.150** | 0.338** | 0.237** | 1.000 |  |  |  |  |  |  |  |
| DBP | 0.154** | 0.148** | 0.294** | -0.161** | 0.308** | 0.246** | 0.787** | 1.000 |  |  |  |  |  |  |
| WBC | 0.076** | 0.117** | 0.314** | -0.221** | 0.056** | 0.211** | 0.141** | 0.153** | 1.000 |  |  |  |  |  |
| M | 0.040** | 0.088** | 0.240** | -0.209** | 0.063** | 0.237** | 0.151** | 0.158** | 0.663** | 1.000 |  |  |  |  |
| B | 0.079** | 0.112** | 0.195** | -0.123** | 0.015 | 0.189** | 0.069** | 0.090** | 0.333** | 0.297** | 1.000 |  |  |  |
| C | 0.069** | 0.044** | 0.074** | -0.054** | .036** | 0.052** | 0.045** | 0.056** | 0.211** | 0.179** | 0.348** | 1.000 |  |  |
| L | 0.123** | 0.162** | 0.274** | -0.149** | -0.010 | 0.213** | 0.071** | 0.082** | 0.618** | 0.423** | 0.269** | 0.160** | 1.000 |  |
| N | 0.029* | 0.058** | 0.246** | -0.193** | .077** | 0.140** | 0.135** | 0.145** | 0.490** | 0.534** | 0.184** | 0.134** | 0.254** | 1.000 |

Abbreviations: TC: total cholesterol; LDL: low-density lipoprotein; TG: triglycerides; HDL: high-density lipoprotein; FBG: fasting blood glucose; UA: uric acid; SBP: systolic blood pressure; DBP: diastolic blood pressure; WBC: white blood cell; M: monocyte; B: basophil; E: eosinophil; L: lymphocyte; N: neutrophil.

Spearman's correlation coefficients were shown in the table, *P<0.05, **P< 0.01.

**Table S2** Intraclass correlation coefficients of metabolic/inflammatory indicators in 2020 and 2021

| Indicators | Coefficients |
| --- | --- |
| Total cholesterol | 0.791** |
| Low-density lipoprotein | 0.793** |
| Triglycerides | 0.781** |
| High-density lipoprotein | 0.820** |
| Fasting blood glucose | 0.631** |
| Uric acid | 0.839** |
| Systolic blood pressure | 0.520** |
| Diastolic blood pressure | 0.683** |
| White blood cell | 0.732** |
| Monocyte | 0.654** |
| Eosinophil | 0.811** |
| Basophil | 0.544** |
| Lymphocyte | 0.752** |
| Neutrophil | 0.691** |

** P<0.01.

**Table S3** Associations between metabolic/inflammatory indicators and thyroid nodules using conditional logistic regression

| Indicators ^a^ |  | Adjusted models ^b^ | | |
| --- | --- | --- | --- | --- |
|  |  | OR (95%CI) | *P-*value | *P*-value ^c^ |
| Metabolic indicators |  |  |  |  |
| TC |  | 1.03 (0.96,1.10) | 0.446 | 0.480 |
| LDL |  | 1.04 (0.97,1.11) | 0.291 | 0.340 |
| TG |  | 1.06 (0.99,1.14) | 0.101 | 0.157 |
| HDL |  | 0.93 (0.87,1.01) | 0.074 | 0.130 |
| FBG |  | 1.09 (1.02,1.17) | **0.011** | **0.026** |
| UA |  | 1.06 (0.97,1.15) | 0.192 | 0.269 |
| SBP |  | 1.14 (1.06,1.22) | **<0.001** | **<0.001** |
| DBP |  | 1.12 (1.04,1.20) | **0.002** | **0.014** |
| Inflammatory indicators |  |  |  |  |
| WBC |  | 1.11 (1.04,1.19) | **0.003** | **0.014** |
| M |  | 1.10 (1.02,1.18) | **0.009** | **0.025** |
| L |  | 1.07 (1.00,1.15) | 0.069 | 0.130 |
| B |  | 1.04 (0.97,1.12) | 0.258 | 0.328 |
| E |  | 0.99 (0.92,1.06) | 0.690 | 0.690 |
| N |  | 1.10 (1.03,1.18) | **0.006** | **0.021** |

Abbreviations: TC: total cholesterol; LDL: low-density lipoprotein; TG: triglycerides; HDL: high-density lipoprotein; FBG: fasting blood glucose; UA: uric acid; SBP: systolic blood pressure; DBP: diastolic blood pressure; WBC: white blood cell; M: monocyte; B: basophil; E: eosinophil; L: lymphocyte; N: neutrophil.

^a^ All indicators were standardized.

^b^ Adjusting age, gender, diabetes, and hypertension.

^c^ False Discovery Rate (Benjamini and Hochberg).


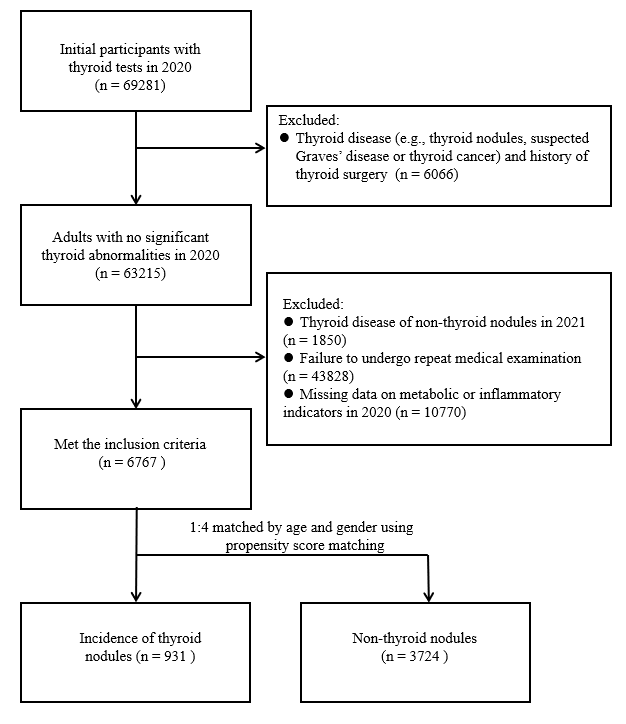
  **Figure S1.** Flow chart


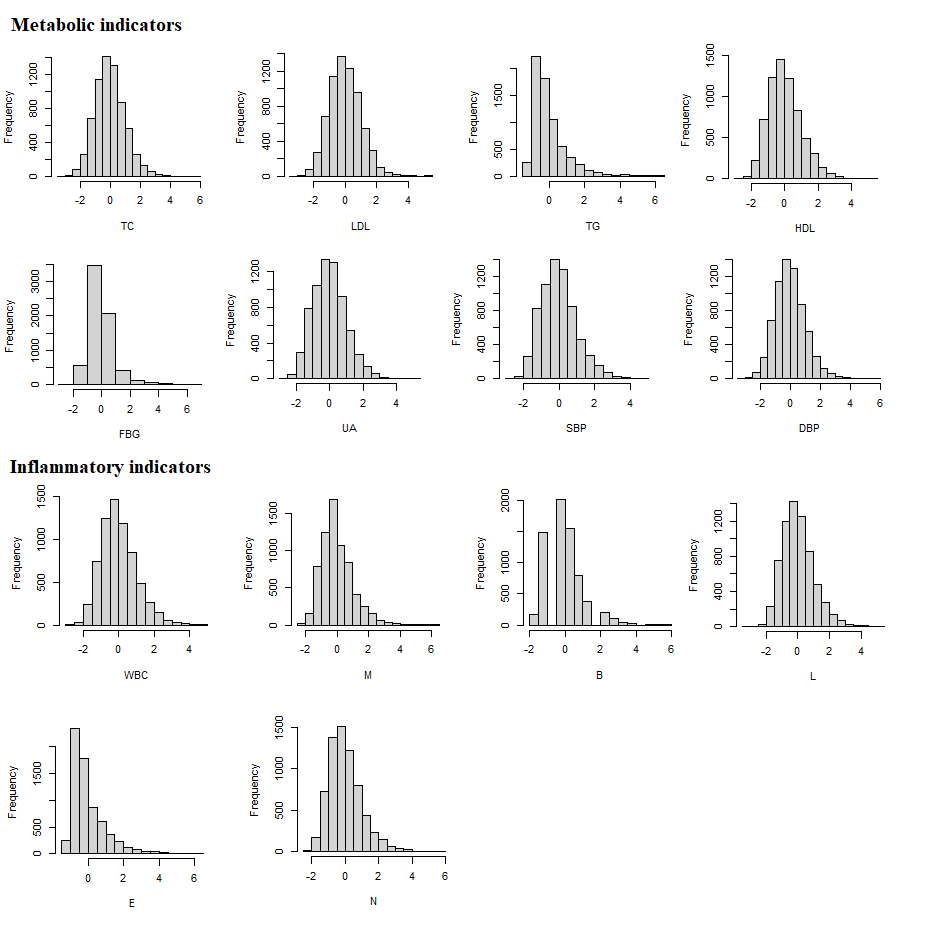
**Figure S2.** The histograms of metabolic/inflammatory indicators


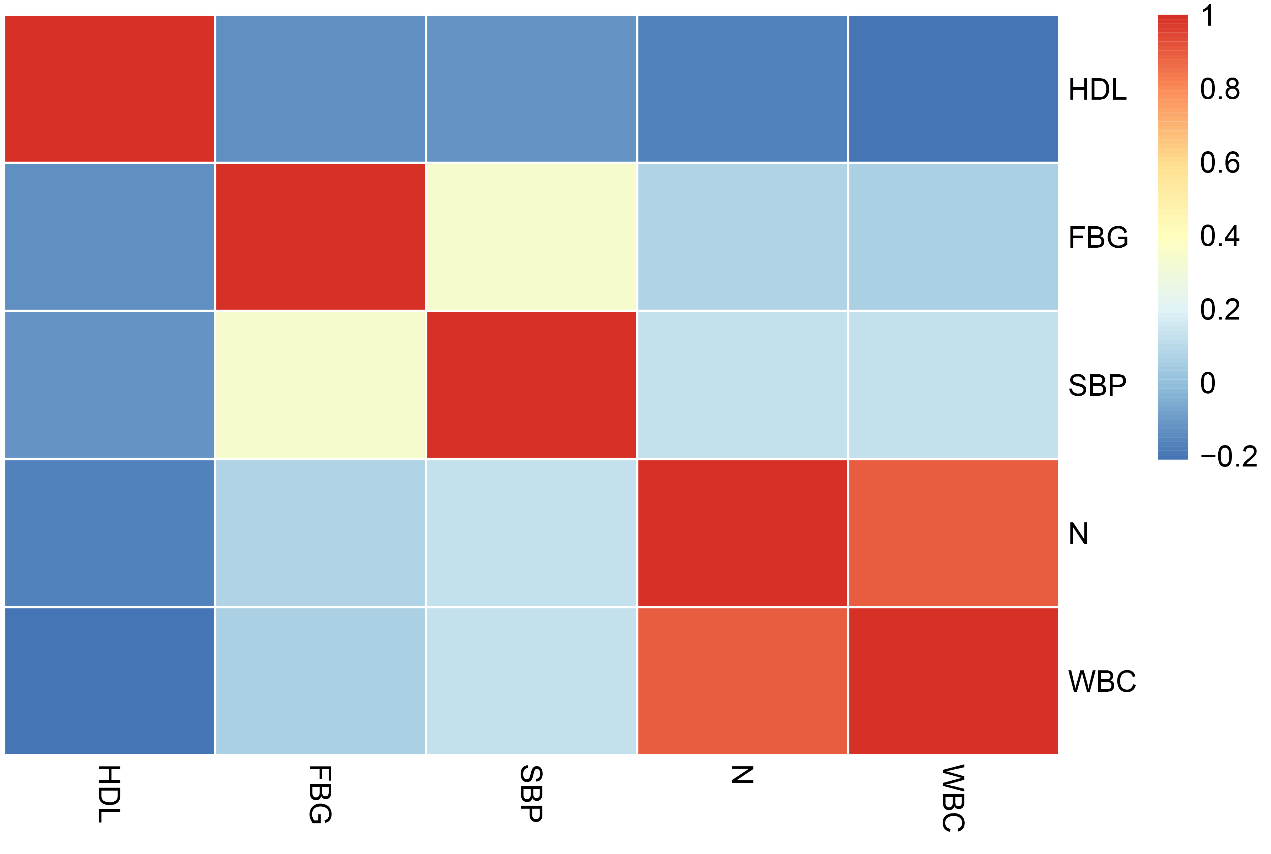
**Figure S3.** Correlations between metabolic/inflammatory indicators using spearman’s correlation analysis

**
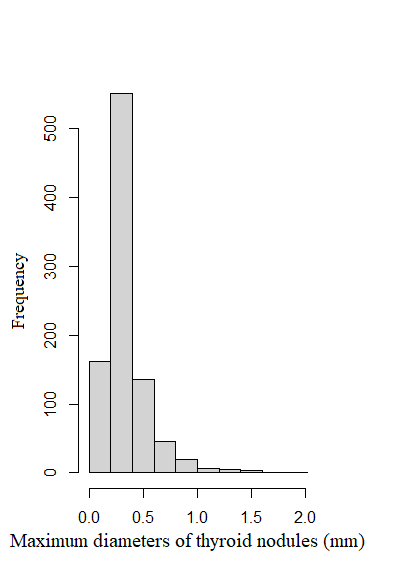
**

**Figure S4.** Maximum diameter of thyroid nodules (mm) in 931 participants


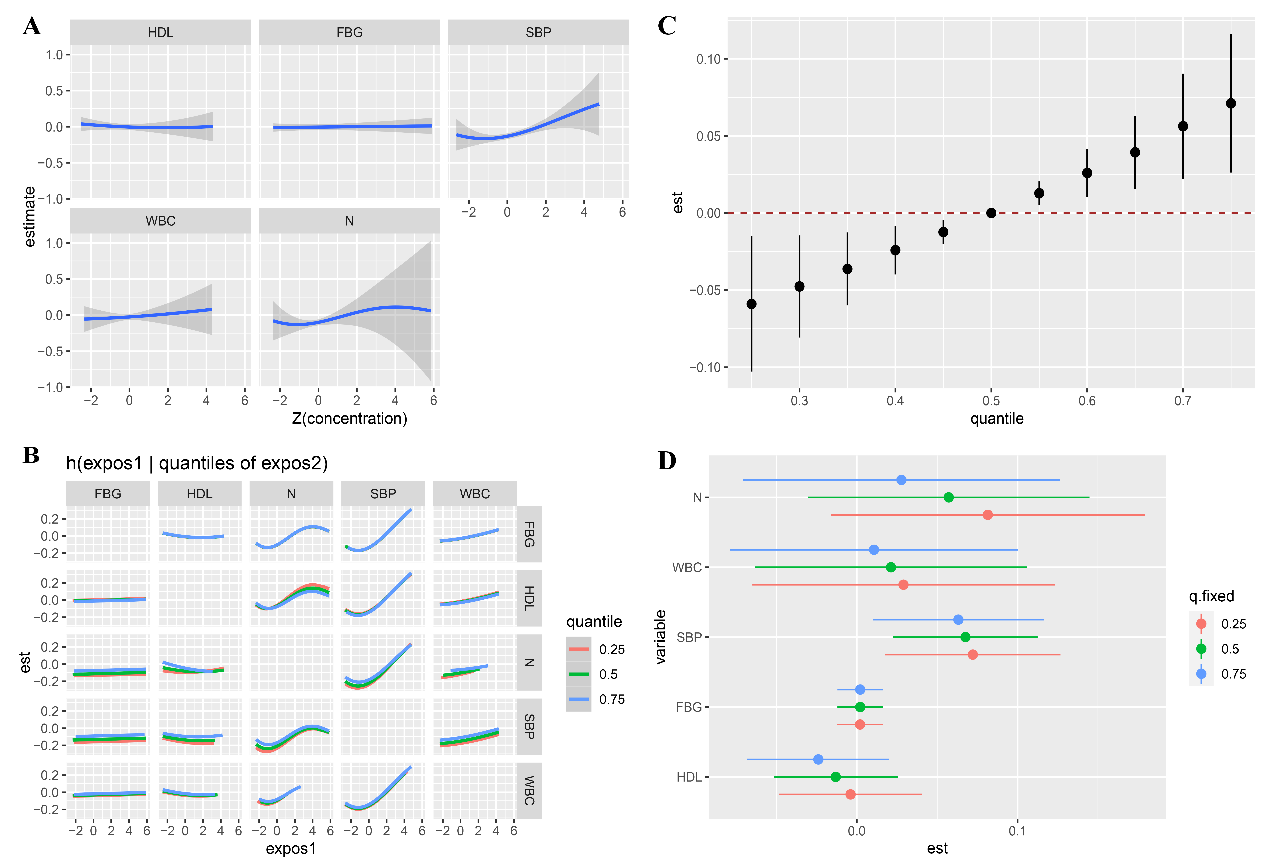


**Figure S5.** Associations between five metabolic/inflammatory indicators and the risk of thyroid nodules in male, estimated using Bayesian kernel machine regression (BKMR). Adjusted variables included age, diabetes, and hypertension.


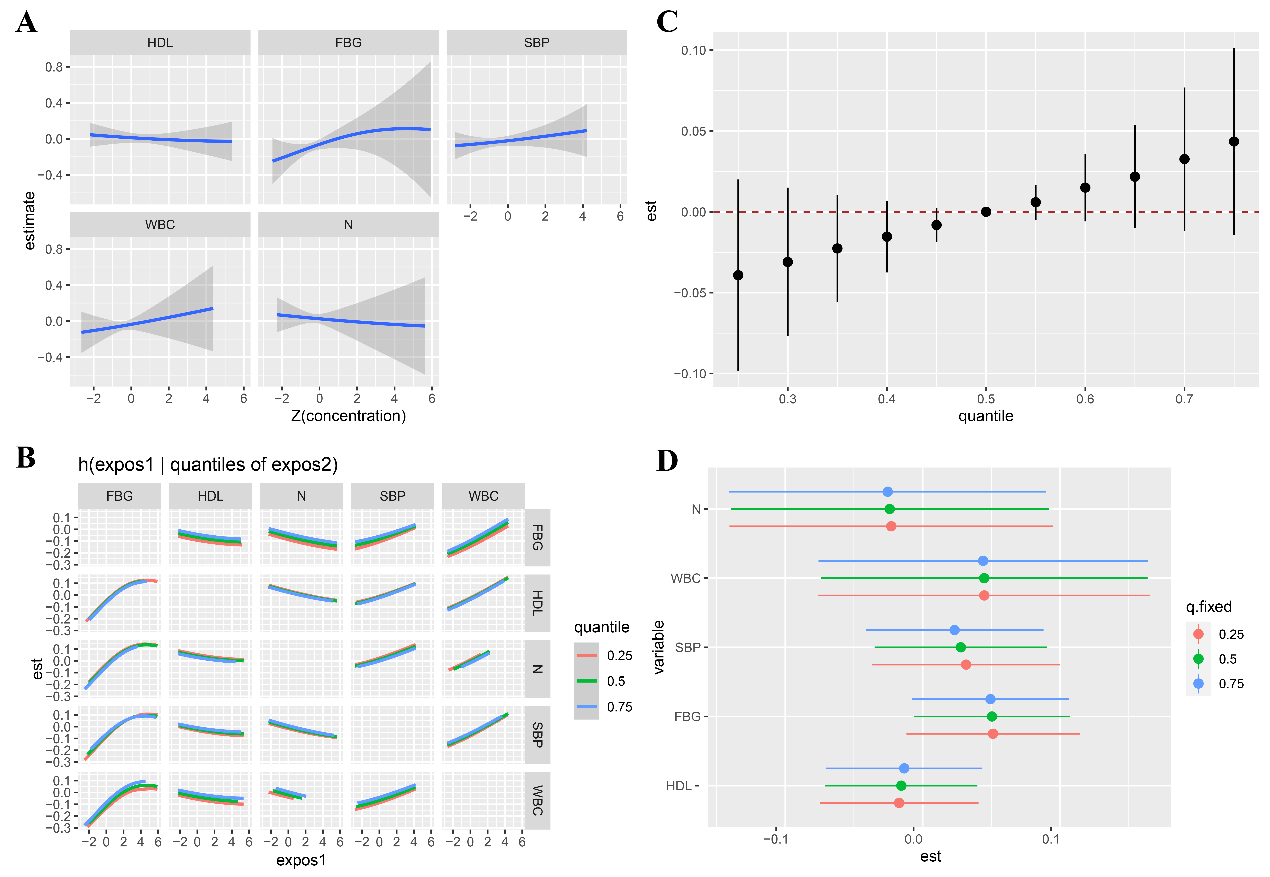


**Figure S6.** Associations between five metabolic/inflammatory indicators and the risk of thyroid nodules in female, estimated using Bayesian kernel machine regression (BKMR). Adjusted variables included age, diabetes, and hypertension.


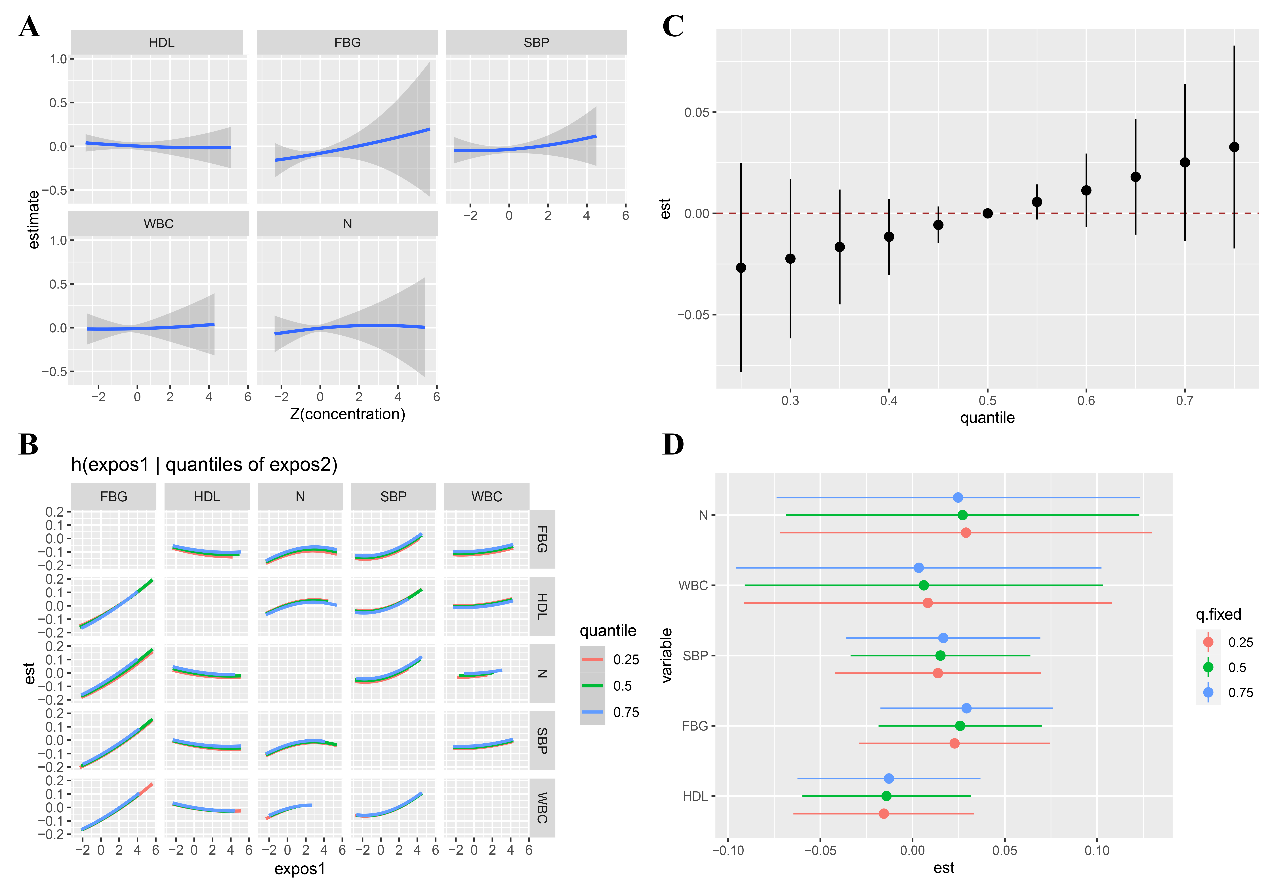


**Figure S7.** Associations between five metabolic/inflammatory indicators and the risk of thyroid nodules in participants with lower age (<40 years old), estimated using Bayesian kernel machine regression (BKMR). Adjusted variables included gender, diabetes, and hypertension.


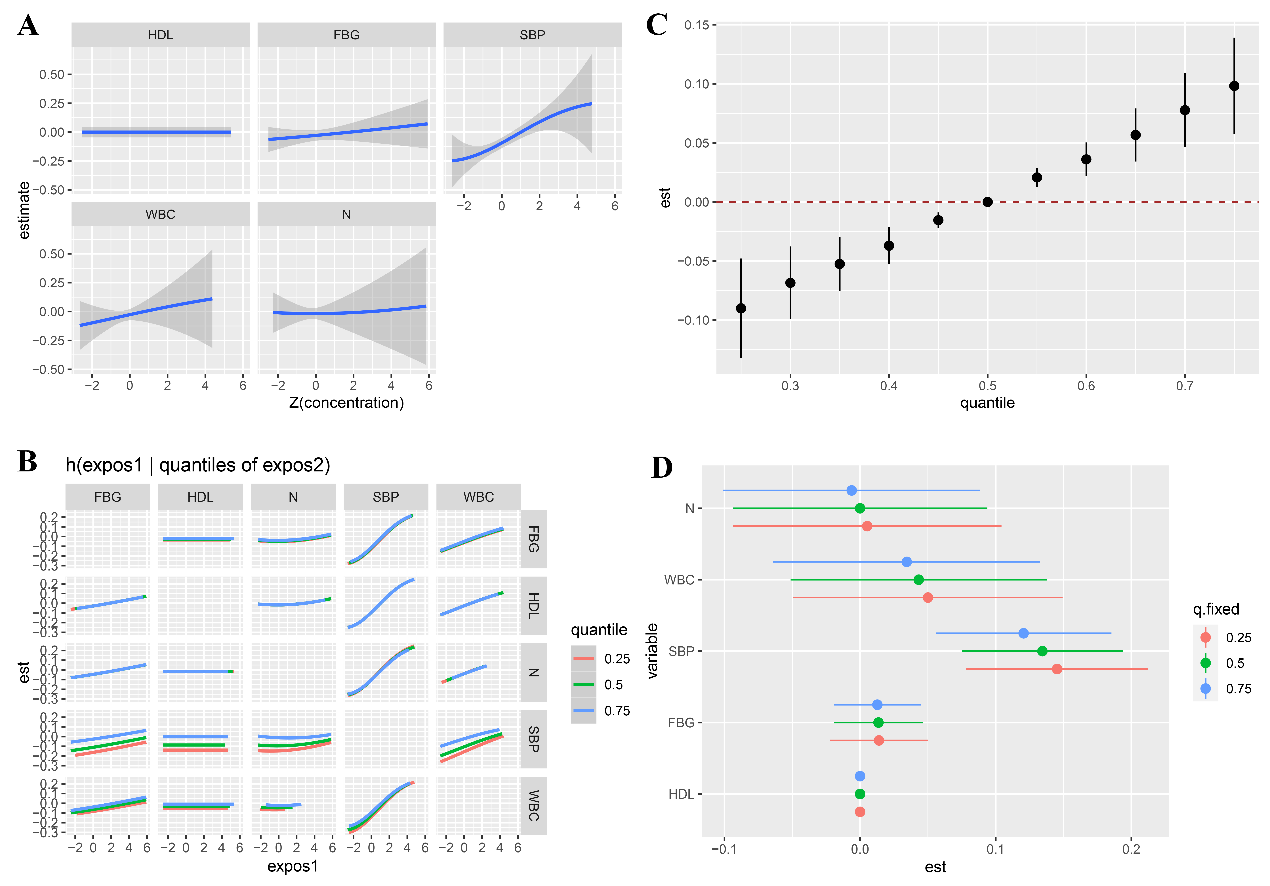
 **Figure S8.** Associations between five metabolic/inflammatory indicators and the risk of thyroid nodules in participants with lower age (≥40 years old), estimated using Bayesian kernel machine regression (BKMR). Adjusted variables included gender, diabetes, and hypertension.


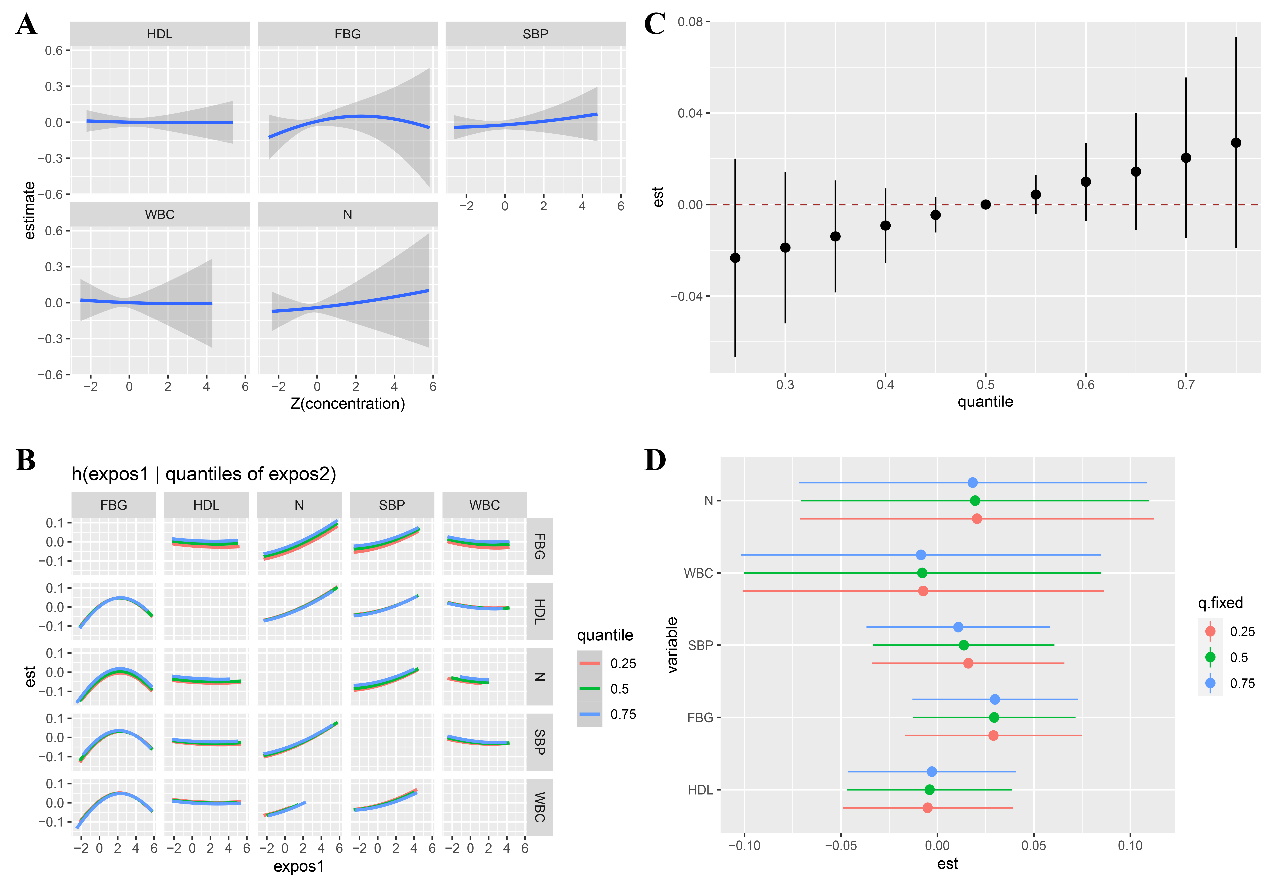


**Figure S9.** Associations between five metabolic/inflammatory indicators and the risk of thyroid nodules in participants with normal body mass index status (18.5–23.9 kg/m^2^), estimated using Bayesian kernel machine regression (BKMR). Adjusted variables included gender, age, diabetes, and hypertension.


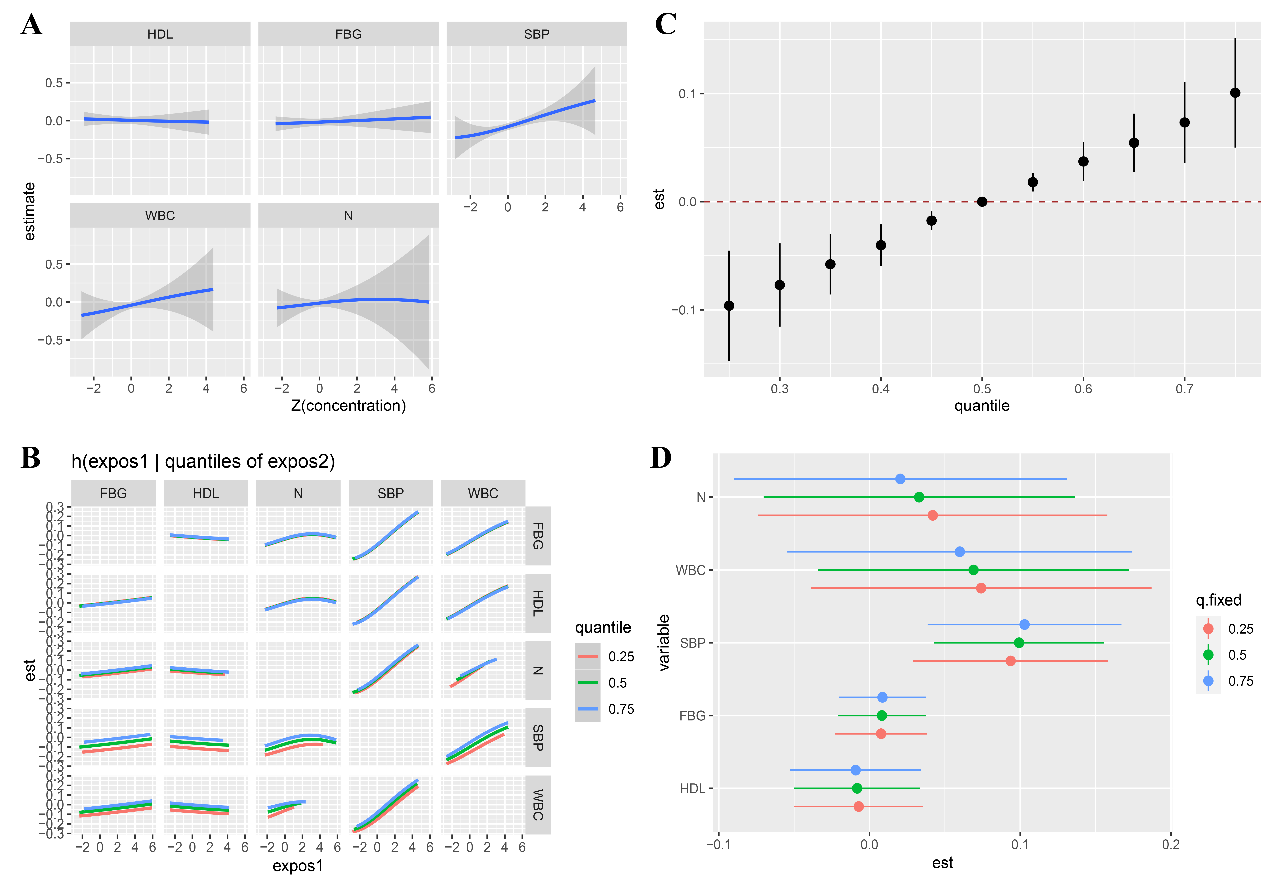
 **Figure S10.** Associations between five metabolic/inflammatory indicators and the risk of thyroid nodules in participants with abnormal body mass index status (<18.5 or >23.9 kg/m^2^), estimated using Bayesian kernel machine regression (BKMR). Adjusted variables included gender, age, diabetes, and hypertension.


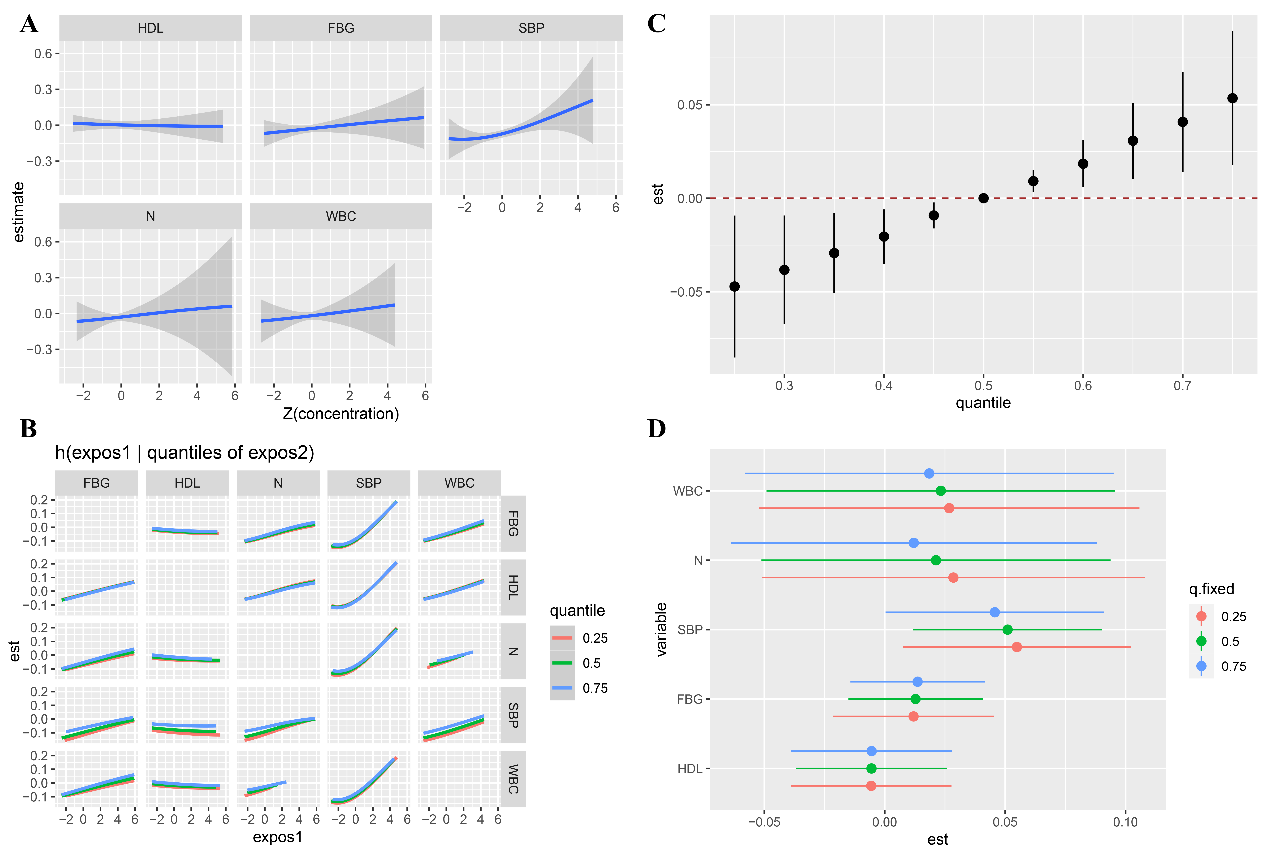


**Figure S11.** Associations between five metabolic/inflammatory indicators and the risk of thyroid nodules in adults, estimated using Bayesian kernel machine regression (BKMR). Adjusted variables included gender, age, diabetes, hypertension, and BMI (continuous).


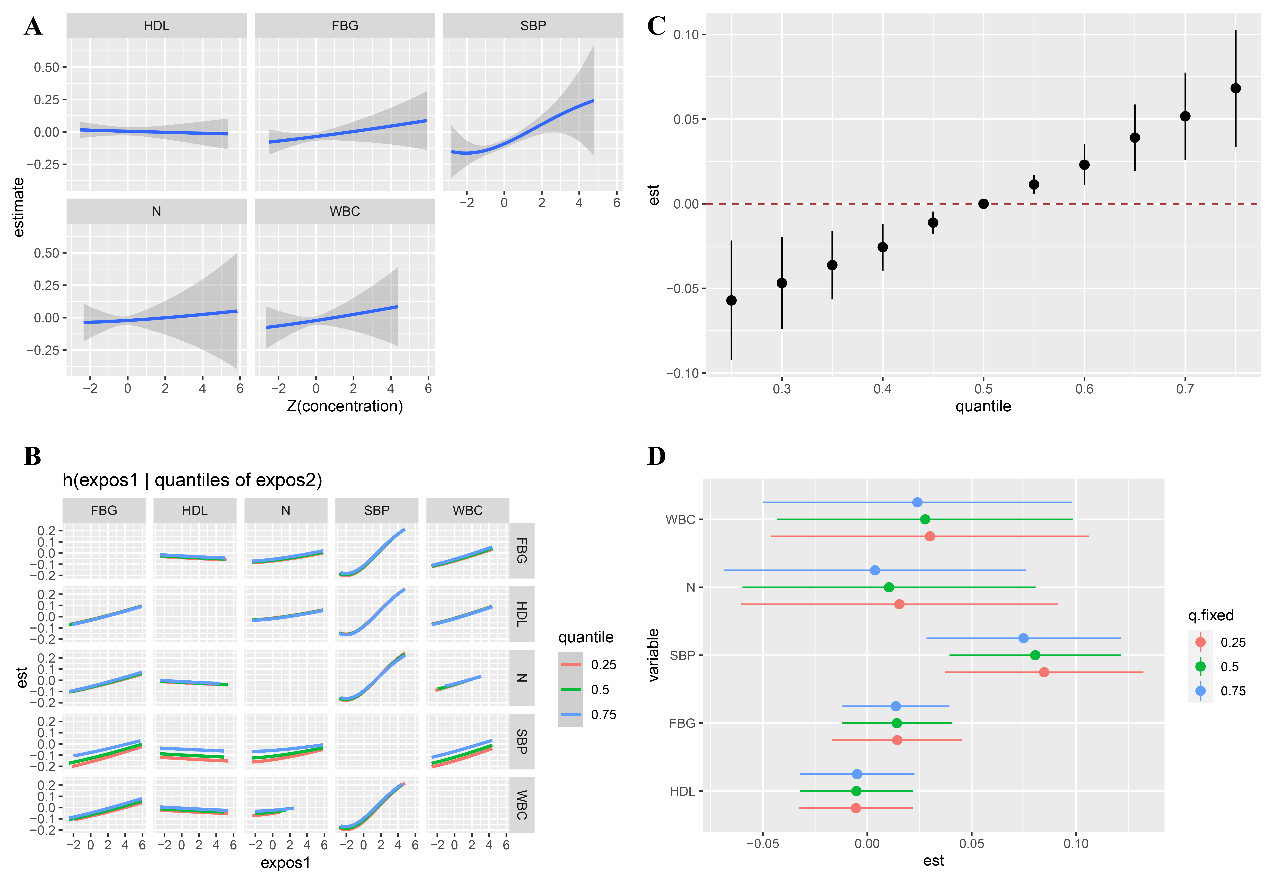
 **Figure S12.** Associations between five metabolic/inflammatory indicators and the risk of thyroid nodules in adults, estimated using Bayesian kernel machine regression (BKMR). Adjusted variables included gender, age, diabetes, hypertension, smoking, and drinking.


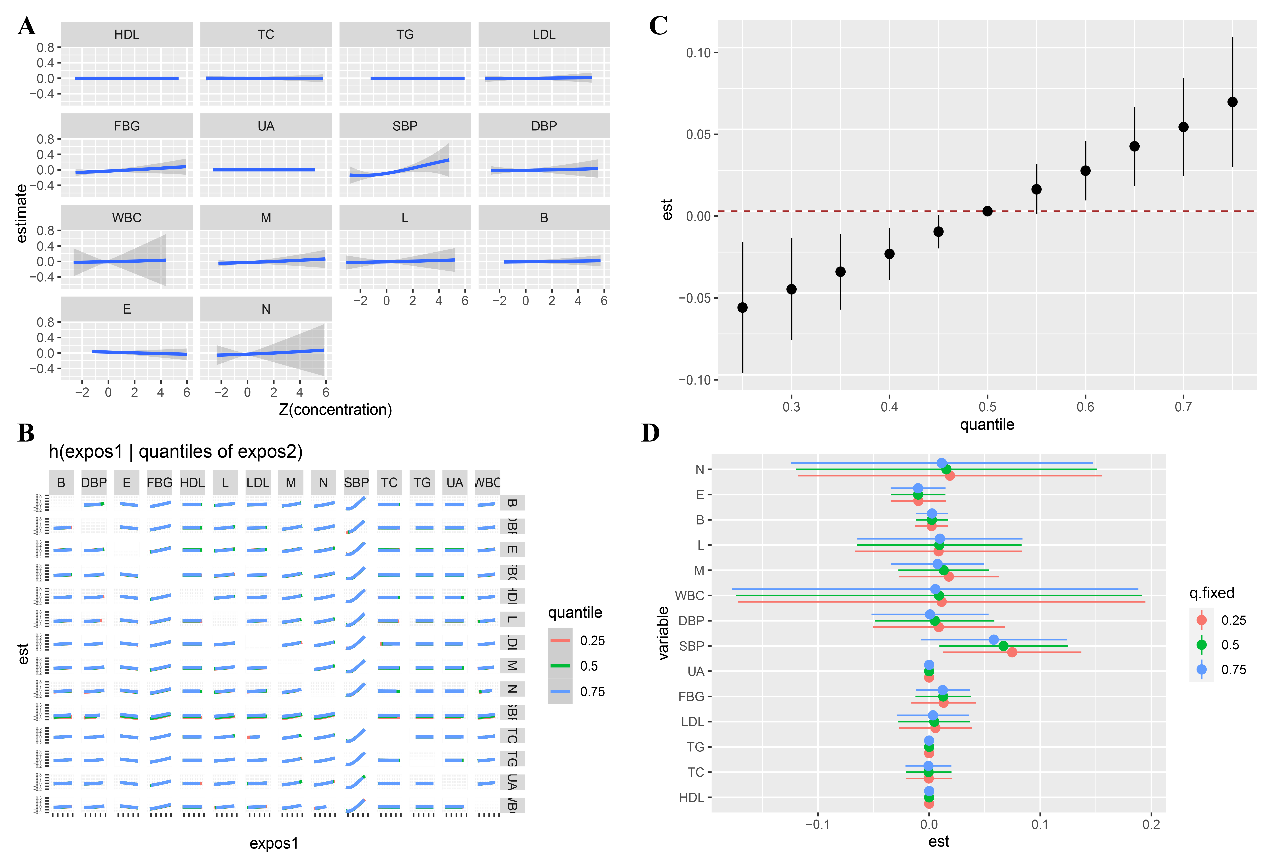
**Figure S13.** Associations between 14 metabolic/inflammatory indicators and the risk of thyroid nodules in adults, estimated using Bayesian kernel machine regression (BKMR). Adjusted variables included gender, age, diabetes, and hypertension.
